# Co-creating care: Involving children, young people, and families in paediatric medical device innovation

**DOI:** 10.3389/fmed.2026.1800244

**Published:** 2026-05-25

**Authors:** Jennifer Preston, Gemma Wheeler, Philippa Howsley, Jessica McNeill, Paul Dimitri

**Affiliations:** 1 Department for Women’s and Children’s Health, University of Liverpool, Liverpool, United Kingdom; 2 National Institute for Health and Care Research, HealthTech Research Centre in Paediatrics and Child Health, Sheffield Children’s NHS Foundation Trust, Sheffield, United Kingdom; 3 National Institute for Health and Care Research, Children and Young People MedTech Co-operative, Sheffield Children’s NHS Foundation Trust, Sheffield, United Kingdom

**Keywords:** child-right’s, co-design, HealthTech, patient and public involvement, participatory design

## Abstract

Innovating in paediatric healthcare requires more than technological advancements; it demands meaningful collaboration with children and young people (CYP) and their families. This practice-focused paper demonstrates how co-creation can be embedded within paediatric medical device development by applying a rights-based approach to guide and inform the involvement activities. Drawing on three illustrative case studies from the National Institute for Health and Care Research (NIHR) HealthTech Research Centre in Paediatrics and Child Health (HRC-PCH), we show how CYP and their families contributed at various stages of innovation, from early concept development to prototype refinement. Each case is examined using Lundy’s Model of Participation and the 3Ps framework (Provision, Protection, and Participation), allowing us to identify practical enablers and barriers to inclusive, ethical, and rights-based involvement. The cases highlight how co-creation can enhance the relevance, usability, and acceptability of paediatric medical technologies while fostering trust, equity, and empowerment among participants. We conclude by outlining actionable, practice-based recommendations for embedding CYP and family involvement within the culture, governance, and day-to-day processes of paediatric innovation. This offers a practical roadmap for teams engaged in research, healthcare, and industry.

## Background

Investing in the health and wellbeing of children and young people (CYP) is widely accepted as a societal priority and a foundation for long-term population health (1). As digital technologies continue to reshape healthcare delivery, diagnostics, and prevention, there is growing global interest in how innovation can address unmet needs in paediatrics. Over several decades, fields such as human–computer interaction (HCI), child–computer interaction (CCI), and participatory design (PD) have demonstrated the creativity and capability of CYP as contributors to technology design, producing rich bodies of work around play, learning, well-being, and self-expression (2–4). These areas emphasise the value of co-creation, agency, and the contextual nature of CYP’s interactions with technology.

Despite this strong academic foundation, the development of clinically integrated, regulated, and scalable digital health solutions for CYP remains limited. Few child-focused technologies advance beyond early prototypes or small feasibility studies to achieve real-world implementation, healthcare commissioning, or approval as regulated medical devices (5, 6). This translational gap is particularly pronounced for CYP with long-term illnesses, disabilities, and neurodevelopmental conditions, as well as those experiencing structural disadvantages. For these groups, issues such as digital exclusion and unmet needs remain prevalent (7, 8). Consequently, progress in clinical paediatric innovation often trails behind broader advances in HCI, creating a persistent gap in equitable solutions for CYP in real-world healthcare settings.

Recognising this challenge, the past decade has seen the emergence of dedicated child health technology networks designed to bridge academic, clinical, and industry expertise. In the UK, the national Technology Innovation Transforming Child Health (TITCH) network and the National Institute for Health and Care Research Children and Young People MedTech Co-operative (NIHR CYP MedTech) have been established to support the development and evaluation of paediatric technologies, collectively leveraging major investments to enhance the capacity for innovation (9). This work has since expanded with the creation of the NIHR HealthTech Research Centre in Paediatrics and Child Health (NIHR HRC-PCH), along with similar initiatives internationally, such as Great Ormond Street Hospital’s Data Research, Innovation and Virtual Environments unit (GOSH DRIVE) and Alder Hey Innovation in the UK, i4KIDS in Spain, and KidsX in the United States. These multi-professional, multidisciplinary centres and networks share a commitment to ensuring that paediatric innovation is responsive to the needs, experiences, and rights of CYP.

### Involvement of children and young people in healthcare technology development

Central to the NIHR HRC-PCH’s approach is the principle of ‘health technology developed for CYP, with CYP’ and ‘no health technology for us, without us’. This aligns with the NIHR’s definition of patient and public involvement (PPI), which emphasises that research and innovation should be carried out ‘with’ or ‘by’ members of the public rather than ‘to’, ‘about’, or ‘for’ them (10). In the context of paediatric innovation, this requires approaches that are not only participatory but explicitly rights-based.

Our ‘work’ with CYP and families is grounded in the 3Ps framework (Provision, Protection, and Participation), which is derived from the United Nations Convention on the Rights of the Child (UNCRC) (11).

- *Provision* involves ensuring that CYP have access to the resources, information, and support necessary for their well-being.
- *Protection* focuses on safeguarding CYP from harm and ensuring ethical, safe practices in their involvement.
- *Participation* emphasises enabling CYP to express their views and ensuring that those views are given due weight in matters that affect them.

These principles are not merely aspirational; they are legal obligations for public authorities in the UK and elsewhere, requiring duty bearers—including researchers, clinicians, designers, funders, and policymakers—to embed CYP rights within decision-making processes (11). A rights-based approach, therefore, provides a robust ethical and practical foundation for paediatric innovation, ensuring that technologies are not only effective but also equitable, acceptable, and in harmony with the experiences and values of CYP.

### Putting rights into practice

Implementing these rights requires interactive partnerships between researchers, designers, PPI practitioners, youth workers, and other stakeholders to conceive, inform, plan, execute, disseminate, and translate research (12). However, involving CYP is complex and multi-dimensional, necessitating attention to four interrelated considerations: (i) the level of participation (i.e., degrees of power-sharing between adults and CYP); (ii) the focus of decision-making (individual or collective); (iii) the model of participation (consultation, collaboration, or child-led); and (iv) clarity about who is meant by ‘children’, recognising the diversity of personal circumstances (age, sex, ethnicity, culture, disability, and social and economic contexts) and the evolving interests and capacities of CYP over time (13).

To be meaningful, all partners need the skills, resources, and structures to participate effectively, and activities should align with agreed good practice standards (14). In addition, the involvement of CYP must be conducted ethically, consistent with UNCRC General Comment No. 12 on the right of the child to be heard (15) (see Table 1). Setting clear aims and roles at the outset supports equitable relationships and helps CYP grow as empowered research partners.

**Table 1:** Nine basic requirements for effective and ethical CYP involvement.

Requirement	Description
Requirement one	Participation is transparent and informative
Requirement two	Participation is voluntary
Requirement three	Participation is respectful
Requirement four	Participation is child-friendly
Requirement five	Participation is inclusive
Requirement six	Participation is inclusive
Requirement seven	Participation is supported by training for adults
Requirement eight	Participation is safe and sensitive to risk
Requirement nine	Participation is accountable

**Figure 1:**
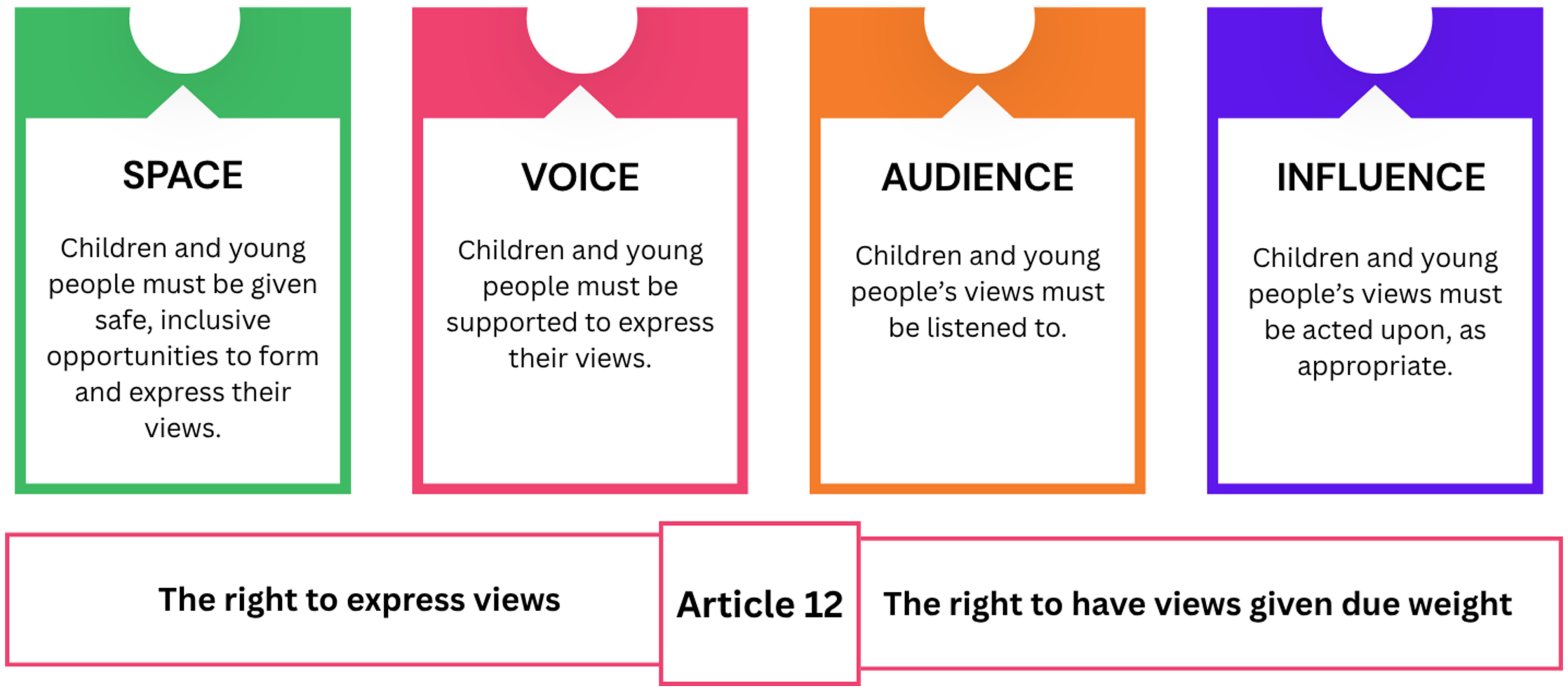
Children and young people’s participation framework informed by the Lundy model. Adapted with permission from “Conceptualising Article 12” by Laura Lundy.

To operationalise these principles, the HRC PRC draws on the Lundy Model of Participation through author developed adaptations, including a conceptual figure (Figure 1) and a participation checklist (Table 2) (16, 17). The model translates Article 12 of the UNCRC into four mutually reinforcing dimensions: “space, voice, audience, and influence”. In practice, the checklist provides a transparent route map from intention to impact, ensuring that CYP’s needs and preferences are acted upon throughout the research process.

**Table 2:** Checklist informed by the Lundy model of participation (17).

**Space**	**Voice**	**Audience**	**Influence**
Children and young people must be given safe, inclusive opportunities to form and express their views	Children and young people must be supported to express their views	Children and young people’s views must be listened to	Children and young people’s views must be acted upon, as appropriate.
How will children and young people be invited to take part?	How will children and young people be supported to share their views in ways that work for them?	Who will listen to the views shared by children and young people?	How will children and young people’s views be used to inform decisions or actions?
What safe and supportive spaces will be available for them to share their views?	What different ways of sharing views will be offered (for example, talking, writing, drawing, or digital activities)?	How will children and young people be told who will see or hear their views?	What plans are in place to feed back to children and young people about what happened next?
What will be done in advance to help all children and young people take part, including those who may face barriers?	What time, information, or preparation will children and young people need to help them express their views?	What steps will be taken to make sure adults involved are prepared to listen respectfully?	How will this be explained if some views cannot be acted on?

The purpose of this article is both conceptual and practice-oriented. Rather than presenting empirical research findings, we draw on three purposively selected examples of involvement with CYP and families to illustrate how rights-based participation can be embedded across the paediatric innovation pathway. These cases cover the entire process from early concept generation to prototype development and refinement, reflecting the diversity of involvement activities undertaken within the NIHR HRC-PCH.

The cases serve as illustrative examples through which we explore the practical application of the 3Ps framework and Lundy’s model of Participation. Analysing the cases through these lenses enables us to identify real-world enablers and barriers to ethical, inclusive, and impactful involvement. Collectively, the examples demonstrate how co-creation can strengthen the relevance, usability, trustworthiness, and equity of paediatric medical technologies.

### Case studies

This section presents the three case studies in detail, demonstrating how CYP and families were involved at different points in the innovation process and how their contributions shaped decision-making and design. The cases were purposively selected to highlight variation in: (i) modes of involvement (all conducted online), (ii) stages of the innovation pathway, and (iii) populations and participant groups engaged.

A concise overview of each case is provided in Table 3, with extended descriptions available in Supplementary files 1–3. Each Supplementary file follows the Guidance for Reporting Involvement of Patients and the Public 2 (GRIPP2: SF) (18) to ensure transparent, comparable, and structured accounts of involvement processes and outcomes. The following section structures our reflections around each Lundy dimension to demonstrate how these principles operate in real-world innovation contexts.

**Table 3:** Summary of NIHR HRC-PCH case studies.

Title and project stage	Context and aims	PPI processes			Impact
		Participants	Recruitment	Methods	
Case Study 1: Young Person Reviewer Workshop (CS1) *[Pre-funding stage]*	The NIHR CYP MedTech Co-operative was awarded funding by the NIHR to support proof-of-concept technology projects aimed at improving CYP’s mental health. This project aimed to gather the views of CYP to help determine which applications should be funded.	19 CYP participated in the workshop. CYP were aged between 12 and 22 years (average age of 17 years). 13 CYP were female, 5 were male, and 1 identified as non-binary.	A recruitment flyer was circulated via the 21 NIHR GenerationR Young People’s Advisory Groups (YPAGs). Interested CYP completed an expression of interest form.	Participants were provided with easy-read versions of the applications before the workshop. During the online workshop, CYP discussed the applications and indicated whether each application should be funded. CYP received a £40 thank-you voucher.	The feedback from CYP helped determine which applications should be funded. The applications ranked highest by CYP aligned with the top-scoring projects from healthcare professional reviewers. CYP provided new insights and useful feedback to project teams whose applications were not successful. A young person who attended the workshop wrote a blog piece about the importance of being involved.
Case Study 2: Nell and the Neonatal Unit (CS2) *[Early-stage prototype]*	Having a newborn sibling admitted to the neonatal unit can be a distressing experience, particularly for older siblings. This project is developing an app to help parents and carers support their older children while they have a newborn in the neonatal unit.	11 participants took part: 6 adults (all female) and 5 children (4 female and 1 male, aged 5–10 years)	Social media and mailing lists were used to recruit relevant participants.	Participants were asked to download and use a beta version of the app with their children before the focus group. The online focus group focused on discussions around the function, usability, and content of the app. Each family received a £20 thank-you voucher.	The focus group had a significant impact on the functionality and content of the app. The success of the focus group encouraged the project team to continue conducting meaningful PPI going forward. Participants enjoyed taking part and felt that their views were listened to. The results of the focus group have also been presented at international conferences.
Case Study 3: Elixir (CS3) *[Late-stage prototype]*	Emergency services are under intense pressure, and new solutions are needed to help triage patients. This project is developing an AI platform to help triage patients contacting 999/111 services, with PPI being used to inform the platform design.	5 parent participants took part in phase 1 workshops (4 female and 1 male), and 5 parent participants took part in phase 2 workshops (3 female and 2 male).	Flyers, social media, and mailing lists were used to recruit relevant participants.	Two phases of online focus groups were conducted. The first focused on how parents and carers would use the AI platform, while the second focused on receiving feedback on the initial prototype. Participants received a £20 thank-you voucher.	The feedback gathered from the focus groups has resulted in substantial changes to the prototype.

### Concept 1: space

Creating a supportive space in a safe environment for person-centred activities is fundamental for CYP and families to express their views in a manner they feel comfortable with.

Within the NIHR HRC-PCH infrastructure, both the skill set of project managers trained in PPI and the remit to encourage and support PPI play a key role in creating a safe ‘space.’ The fact that the NIHR HRC-PCH team is hosted by a children’s hospital in the UK National Health Service (NHS) ensures they are well trained in ethics, data protection, and online safety. For the teams leading CS2 and CS3, this was their first experience of conducting PPI work. Therefore, the role of the NIHR HRC-PCH was to guide and upskill them through this process, so that they can apply these skills independently to create a safe ‘space’ in future projects.

A key challenge across all the case studies presented, and in PPI more broadly, is taking steps to ensure that all eligible CYP and/or families are able to take part, avoiding the exclusion of diverse and underserved populations. To maximise recruitment for the presented case studies, PPI activities were conducted online. Online involvement activities have many benefits and often reduce many of the barriers faced by CYP and their families when invited to take part in PPI activities, such as the time and monetary costs associated with travel and participation.

However, recruitment methods for all PPI activities must be considered in relation to equality, diversity, and inclusion. Individuals who sign up to participate in PPI activities via digital methods (e.g., social media) may be more familiar with involvement and research activities in general and, as such, may only provide one perspective. Moreover, online activities may automatically exclude CYP who experience digital poverty and do not have access to the necessary technologies or data required to participate. Despite our efforts to maximise diversity, the majority of participants involved in CS2 and 3 were female (particularly in parent/carer groups) and of White British ethnicity. Consequently, several underserved groups were not represented in these projects. Although this is a common limitation in many PPI and research activities, further research is urgently needed to improve diversity across all PPI activities. Critically, innovations that do not respond to and accommodate underserved populations risk perpetuating exclusion and widening existing health inequalities (19).

Partnering with existing groups and infrastructure that support CYP’s involvement, such as charities and youth groups, can help recruit diverse voices. For example, recruitment for the ‘Young Person Reviewer Workshop’ (CS1) was facilitated through the GenerationR Alliance Young People’s Advisory Groups (YPAGs) (20). These YPAGs are funded by the NIHR, NHS, or partner organisations (i.e., higher education institutions, charities, and so on) and support the design and delivery of paediatric health research in the UK. Recruiting through this network enabled the project to reach a broad geographical area and leveraged the existing trust and long-term relationships between CYP and the leaders of each YPAG. This approach was extremely effective, engaging CYP from a wide range of backgrounds and age groups.

Interestingly, in each of the case studies presented, participants demonstrated awareness of, and empathy for, the experiences and needs of other groups of CYP who were not present in the workshops. Indeed, participants in the ‘Elixir’ project (CS3) raised concerns about how other groups of CYP, such as older generations and those who come from lower socioeconomic backgrounds, may feel when interacting with the new technology platform. They suggested that the platform should be considered complementary to existing service pathways rather than replacing them.

Various techniques can help address representation gaps by making the participation space more accessible. In CS1, the use of personas (fictional prompts to elicit alternative perspectives) encouraged CYP to prioritise social justice, equitable access to healthcare, and issues such as data privacy.

In summary, enablers for creating a ‘space’ for meaningful involvement include appropriate ‘provision’ (from the 3Ps model, e.g., via networks such as the NIHR HRC-PCH) to catalyse new ways of working for teams without prior involvement experiences. This requires the more inexperienced teams to be included in the planning and delivery of involvement activities alongside organisations with PPI experience, such as the NIHR-PCH, to ensure they are upskilled and prepared to facilitate future activities. Linking with established young persons’ networks (such as the YPAGs) was also a key enabler for supporting diverse recruitment. However, despite efforts to increase diversity in PPI activities, there is still a significant amount of work to be done, and it is important to underscore that digital engagement methods (as used in the case studies above) can privilege certain demographics.

With this in mind, as we move forward as the NIHR HRC-PCH, we will work to become leaders in equality, diversity, and inclusion within the field of child health technology. Achieving this will require strong partnerships and the adoption of evidence-informed approaches, such as the Minimum Digital Living Standard, which seeks to address digital inequalities experienced by households across all four UK nations (21). The NIHR HRC-PCH will also develop its own initiatives, including robust systems for monitoring CYP and family involvement, enabling the tracking of equality, diversity, and inclusion across all centre activities. Importantly, we also aim to lead through our education programme by developing a Knowledge Centre, an open-access repository of resources to support learning in paediatric health technology, and through the Elevate2Innovate (E2I) programme, which provides a comprehensive training pathway for early-career researchers.

### Concept 2: voice

Regardless of the involvement activity or stage, tailored communications that meet the needs of CYP (e.g., age- and developmentally appropriate information) are required to facilitate the expression of their views. For instance, the team leading the involvement process for CS1 created a resource pack that was sent to CYP 2 weeks before the workshop, which included a shortened, simplified summary of the funding applications they would review, a glossary of key terms they might encounter, and a list of questions they would be asked in the workshop breakout rooms. As a result, CYP were ‘equipped’ with the same knowledge as professional reviewers and were able to enter the involvement ‘space’ on an equal footing. Similarly, the project team involved in CS2 also shared access to an early prototype of the application before the workshops. In both cases, participants were more empowered to form their own ‘voice’ because this provision extended the ‘space’ of the workshop in terms of both *time* (it can be more empowering for participants to be able to reflect on what they would like to share in advance) and *context* (these reflections can be informed by real-world home environments and routines).

Providing a range of ways for CYP and families to choose how they want their voices to be heard has helped create supportive involvement spaces. For example, across the cases presented, participants were encouraged to choose whether they would like to have their cameras on or off and to contribute via their microphone, the video call chat function, and/or on-screen emojis. This, in turn, helped reduce power imbalances in the involvement ‘space’ by ensuring it was adaptable to a range of participation needs and preferences.

Although the enablers described above (such as providing information in advance and allowing CYP to choose the way(s) in which they want to participate) received anecdotal positive feedback from those who took part, no work was carried out to formally assess how effective these methods were, and CYP were not invited to share feedback or ideas to improve our approach. Therefore, this may indirectly create barriers to meaningful involvement in the future. Within the NIHR HRC-PCH, our priority will be to develop tools and methods that support CYP in self-reflecting on their experiences, to establish systematic processes for evaluating and reporting the impact of their involvement across all activities, and to co-create new frameworks and methodologies that enable meaningful involvement.

### Concept 3: audience

The ‘audience’ element of this model focuses on ensuring that CYP’s views are communicated to someone who has the responsibility to listen, the authority to make decisions, and the power to effect change. In many instances within the NIHR HRC-PCH portfolio, this has involved working directly with project teams (as described in the case studies), as well as lobbying funders and policymakers to recognise the value of incorporating the views of CYP and families and to embed these within their culture, structures, and processes. This requires building relationships with all stakeholders (i.e., sponsors, funders, regulators, etc.) who hold the ‘power’ to incorporate CYP and families’ views, educating them about the benefits of involvement, and sharing the outcomes and impact of involvement practice on both the research and CYP and/or parents who get involved.

Feedback is a key motivational factor for CYP and family involvement as it shows how their input is shared, with whom, and the impact it has on the outcomes of the project. In some cases, feedback was provided relatively quickly via an email update. For example, the CYP involved in CS1 were informed within a few weeks of their workshop about which projects were funded based on their reviews, alongside the reviews of professionals. However, in other instances, CYP may be invited to participate in a longer project, and due to the nature of research and innovation in healthcare, progress can be (necessarily) slow. This, in turn, can delay feedback or hinder CYP’s understanding that their involvement is contributing to a larger picture. This was true for CS3 and, on reflection, may have benefited from smaller, regular updates on impact rather than planning to provide CYP with a final ‘impact report’ at the end of the project. Moreover, in cases such as CS2, families can be asked to contribute to a ‘Proof of Concept’ project, where the aim is not to develop a final project, which again requires clear communication to avoid disappointment.

With the above in mind, it is imperative to allocate time and space to manage the expectations of all stakeholders in PPI activities, communicate exactly how CYP are contributing to the project and the wider research landscape, and consider more regular—and potentially more engaging—forms of communicating impact.

Understanding the impact of involvement activities requires clear processes to systematically collect and analyse feedback from all participants and to provide timely feedback, which requires regular communication among all stakeholders. The NIHR HRC-PCH will endeavour to develop systematic processes to improve feedback, reporting, and communication between project teams and PPI participants.

### Concept 4: influence

‘Influence’ refers to ensuring that CYP’s views are taken seriously and acted upon (where appropriate), as well as making sure CYP are provided with feedback explaining the reasons for the decisions taken.

The presented case studies illustrate varying degrees of influence, which can be affected by the stage at which CYP and families are involved. For example, CYP and families involved at an early stage of the research were able to shape research priorities by informing which funding applications should be supported in CS1 and inform significant design changes affecting the usability, appearance, and content of the app in CS2. In CS3, CYP and families were involved in reviewing a later-stage prototype, which led to minor tweaks to the app design rather than substantial changes. However, the research team emphasised the important role this involvement played in ‘de-risking’ the new technology, saving money, and potentially saving lives. In both cases, the research teams reported the influence of involvement in terms of the more ‘human’ considerations that are vital to technology development, such as perceived trust in an innovation or system, and the thoughts and emotions that CYP and families might experience in a given situation.

Meaningful involvement can also influence research beyond the current project. For example, families involved in CS3 suggested new, specific contexts in which the innovation could be adapted and applied, drawing on their experiences of the wider healthcare landscape. Moreover, in CS2, families were able to envision the long-term development of the innovation, suggesting other storylines and characters to extend its benefits to more people—for example, addressing language barriers, religious and cultural differences, and being inclusive of non-nuclear family dynamics.

Influence should be understood as multi-directional, shaping not only the project but also CYP and families. In CS2, several participants reflected that their involvement had a direct positive effect at home, opening communication channels that had previously been hard to access. The workshop explicitly explored how the innovation might support parent–child dialogue during emotionally challenging periods. Both CYP and parents recognised common challenges from their lived experiences and left feeling better equipped to navigate difficult conversations with greater ease. Beyond addressing an unmet healthcare need at the population level, meaningful involvement can benefit participants in the moment by providing a safe, facilitated space for reflection and communication. Although reimbursement (e.g., vouchers) is commonly used to acknowledge time, involvement processes that intentionally consider ‘space’, ‘voice’, and ‘audience’ can be influential and intrinsically rewarding. Across the cases, CYP and families described enjoying contributing to something that would help others. They valued not only being listened to but also listening to the experiences of their peers.

## Discussion

### Regulatory science implications for paediatric medical device co-design

Integrating co-creation with CYP and families into paediatric medical device development carries significant implications for regulatory science, particularly in relation to early-stage evidence generation, human factors engineering, risk identification, and equitable implementation. Across the three case studies, CYP participation generated forms of user-centred evidence that regulators increasingly view as essential for developing safe, effective, and trustworthy technologies.

CYP and family involvement enhanced early problem definition, usability insights, and anticipatory risk identification, each a core component of contemporary regulatory pathways. In Case Study 1, for example, providing CYP with accessible, easy-read application summaries before the workshop enabled meaningful participation in prioritisation decisions, directly shaping which applications were selected for funding. This early confirmation of need, relevance, and acceptability aligns with regulatory expectations that device development be rooted in user-defined requirements. Similarly, in Case Study 2, CYP feedback significantly influenced the functionality and content of the app, while Case Study 3 surfaced concerns about how different groups might use an AI-enabled triage tool, including potential barriers for families living in socioeconomically disadvantaged circumstances. These contributions reflect the human factors and accessibility considerations central to regulatory evaluation.

CYP and family involvement also strengthened risk mitigation and implementation evidence. The Case Study 3 team explicitly described involvement as a means of “de-risking the new technology; saving money and potentially saving lives”, illustrating how lived experiences can help identify unintended consequences, safety issues, and trust barriers early in the development process. At the same time, the manuscript acknowledges key equity limitations: Online involvement activities may inadvertently exclude CYP experiencing digital poverty, and participants in case studies 2 and 3 were predominantly White British families. These gaps highlight the regulatory requirement to demonstrate equitable safety, usability, and performance across diverse populations. The NIHR HRC-PCH’s commitment to approaches such as the Minimum Digital Living Standard and to building robust systems for monitoring CYP and family involvement provides an important foundation for generating the equitable implementation evidence that regulators increasingly expect.

Taken together, these findings indicate that co-design is not only a rights-based obligation but also a regulatory asset, producing the human-centred evidence required to ensure that paediatric medical devices are safe, effective, and trusted by the CYP and families who rely on them.

In summary, key enablers of effective co-design include involving CYP and families as early as possible, creating space for unexpected insights to emerge, and adopting a broad view of reciprocity that recognises the value of ensuring involvement activities provide direct benefits to CYP and families. A recurring barrier, however, is the time, confidence, and skill required by teams to embed these practices meaningfully. To address this, the NIHR HRC-PCH is developing a suite of educational resources, including training materials, best-practice principles, and case examples illustrating the tangible impact of CYP and family involvement. This work aims to strengthen the future workforce and support a sustained, CYP rights-based approach to child health technology development and beyond.

## Conclusion

In general, it is now accepted that the voices of CYP and their families must be heard and listened to in matters that concern them. This applies not only to research and innovation in child health technologies but also to healthcare more broadly. The practice of involving CYP and families as active partners in research needs to be meaningful for all participants. This requires being open about the ‘messy’ reality and the practical challenges of involvement, so that we (as a community) can develop our involvement practices.

We suggest that future involvement should be grounded in a rights-based approach, providing a robust basis for planning, decision-making, and action. The Lundy Model offers a practical structure for designing these activities and for systematically identifying potential gaps before engaging with CYP and families.

By using this model to reflect on a range of case studies from the NIHR HRC-PCH portfolio, we present a series of enablers and barriers to PPI in child health technology development (summarised in Table 4), as well as ‘lessons learned’ to support the development of meaningful CYP and family involvement practices.

**Table 4:** Summary of enablers and barriers to PPI in child health technology development, using Lundy’s model of participation to reflect on case studies 1–3.

Domain of Lundy’s model of participation (8)	Enablers of PPI in child health technology development	Barriers to PPI in child health technology development
Space	Project team has the necessary PPI skills to create a safe space, and provision is available to upskill new team members. Partnering with existing groups and infrastructure that support CYP’s involvement, such as charities and Young People’s Advisory Groups. Including PPI activities that consider the experiences of people not ‘in the room,’ such as Personas.	Recruitment methods that privilege or are only accessible to certain demographics.
	Conducting PPI online can be an enabler for some (reducing time and travel costs) but a barrier for others (i.e., those experiencing digital poverty).	
Voice	Providing age- and developmentally appropriate information in advance of PPI activities. Providing a range of options for CYP and families to choose how they want their voices to be heard.	Failing to invite feedback from CYP and families on their experiences of the PPI process/activities and on how they could be improved.
Audience	Building relationships with all stakeholders who hold the ‘power’ to act on CYP and families’ input. Clearly communicating and managing expectations regarding project timescales and outcomes.	Failure to provide feedback to CYP involved in research and innovation in a timely manner, or at all.
Influence	Involving CYP and families early in the project. Allowing space for unexpected outcomes within involvement activities. Taking a holistic view of reciprocity and considering how CYP and families could benefit from participation.	Dedicated time is needed to conduct PPI meaningfully. Developing the skills needed to plan and conduct meaningful PPI.

Moving forward, this article also serves as a catalyst for continuous improvement, highlighting ways in which we will embed involvement into the culture, structure, and processes of the next stage of our journey as the NIHR HRC-PCH, while offering guidance for others.

## Data Availability

Full datasets related to the technologies generated through the case studies described in this paper are not publicly available, due to preservation of intellectual property while the projects are ongoing. However, datasets related to the PPIE approaches used in the case studies are available in Supplementary information files 1–3.

## References

[1] Clark H Coll-Seck AM Banerjee A Peterson S Dalglish SL Ameratunga S . A future for the world's children? A WHO–UNICEF–*lancet* commission. Lancet. (2020) 395:605–58. doi: 10.1016/s0140-6736(19)32540-1, 32085821

[2] Druin A. The role of children in the design of new technology. Behav Inf Technol. (2002) 21:1–21.

[3] Read JC Markopoulos P. Child–computer interaction. Int J Child-Comput Interact. (2013) 1:2–6. doi: 10.1016/j.ijcci.2012.09.001

[4] Yarosh S . "Supporting parent–child communication in everyday life". In: CHI Conference Proceedings. Cambridge: ACM (2011)

[5] Duffy S Krishnan A Yazdi Y Quan X Hughes M Marsal AL . The challenges and opportunities in Pediatric medical device innovation: monitoring devices. Ann Thorac Surg. (2025) 120:428–39. doi: 10.1016/j.athoracsur.2024.11.034, 39716532

[6] Mills N Howsley P Bartlett CM Olubajo L Dimitri P. Overcoming challenges to develop technology for child health. J Med Eng Technol. (2022) 46:547–57. doi: 10.1080/03091902.2022.2089254, 35730496

[7] Hollis C Falconer CJ Martin JL Whittington C Stockton S Glazebrook C . Annual research review: digital health interventions for children and young people with mental health problems - a systematic and meta-review. J Child Psychol Psychiatry. (2017) 58:474–503. doi: 10.1111/jcpp.12663, 27943285

[8] Available online at: https://healthinnovationeast.co.uk/wp-content/uploads/2025/03/07.-Rapid-evidence-review-CYP-digital-exclusion-Jan-24_FINAL.pdf (Accessed January 7, 2026).

[9] National Institute for Health and Care Research. NIHR Children and Young People’s Health Research Centre five-year Report 2018–2023. Available online at: https://hrc-children.nihr.ac.uk/wp-content/uploads/2024/05/2023-Five-Year-Report-web.pdf (Accessed December 22, 2025).

[10] National Institute for Health and Care Research. Briefing notes for researchers - public involvement in NHS, health and social care research. (2021). Available online at: https://www.nihr.ac.uk/documents/briefing-notes-for-researchers-public-involvement-in-nhs-health-and-social-care-research/27371. (Accessed March 27, 2024).

[11] United Nations Convention on the Rights of the Child. (2009). Available online at: https://www.ohchr.org/en/instruments-mechanisms/instruments/convention-rights-child (Accessed February 1, 2024).

[12] Wheeler G Mills N Ankeny U Howsley P Bartlett C Elphick H . Meaningful involvement of children and young people in health technology development. J Med Eng Technol. (2022) 46:462–71. doi: 10.1080/03091902.2022.2089252, 35852341

[13] Sinclair R. Participation in practice: making it meaningful, effective and sustainable. Child Soc. (2004) 18:106–18. doi: 10.1002/chi.817

[14] National Institute for Health and Care Research. Going the extra mile: improving the nation’s health and wellbeing through public involvement in research. (2015). Available online at: https://www.nihr.ac.uk/documents/about-us/our-contribution-to-research/how-we-involve-patients-carers-and-the-public/Going-the-Extra-Mile.pdf (Accessed March 27, 2024).

[15] United Nations Convention on the Rights of the Child. General Comment No. 12: The right of the child to be heard. (2009)

[16] Lundy L. Voice’ is not enough: conceptualising article 12 of the United Nations convention on the rights of the child. Br Educ Res J. (2007) 33:927–42. doi: 10.1080/01411920701657033

[17] Department of Children and Youth Affairs. National Strategy on Children and Young People’s Participation in Decision-Making 2015–2020. Dublin: Government Publications (2015).

[18] Staniszewska S Brett J Simera I Seers K Mockford C Goodlad S . GRIPP2 reporting checklists: tools to improve reporting of patient and public involvement in research. BMJ. (2017) 358:j3453. doi: 10.1136/bmj.j3453, 28768629 PMC5539518

[19] Tooman TR Frost H Adams R Anand A Aujla N Bareham B . Excluded by design: a qualitative study of inequalities in health and social care innovation. Int J Equity Health. (2026) 25:22. doi: 10.1186/s12939-025-02751-5, 41519736 PMC12828945

[20] GenerationR Alliance. (2014). Available online at: https://generationr.org.uk/generationr-alliance/ (Accessed December 12, 2025).

[21] Yates S Hill K Blackwell C Davis A Padley M Stone E . A Minimum Digital Living Standard for Households with Children: Overall Findings Report. [report]. London: University of London (2024).

