# Supplementary material for: Co-creating care: Involving children, young people, and families in paediatric medical device innovation
Source: Front Med (Lausanne). 2026 May 25;13:1800244. doi: 10.3389/fmed.2026.1800244 (PMC13244872; doi:10.3389/fmed.2026.1800244)
Supplement: Supplementary file 1 [file Data_Sheet_1.PDF]

## Supplementary Material

The supplementary material for this publication are as follows:

- Supplementary File 1: GRIPP2 Short form for Case Study 1: Young Person's Reviewer Workshop
- Supplementary File 2: GRIPP2 Short form for Case Study 2: Nell and the Neonatal Unit
- Supplementary File 3: GRIPP2 Short form for Case Study 3: Elixir

## Supplementary File 1: GRIPP2 Short form for Case Study 1: Young Person's Reviewer Workshop

| Section and topic | Item |
|---|---|
| 1: Aim(s) of the PPI | To consider the views of CYP alongside professionals in the review of applications to Proof of Concept funding in the context of innovations to support mental health. Applications could address one or two key themes:<br><br>The mental health of CYP with long-term conditions<br>The mental health of family members of CYP with long-term conditions (particularly parents/caregivers of children in the neonatal unit) |
| 2: Methods<br><br>Provide a clear description of the methods used for patient and public involvement (PPI) in the study | A recruitment flyer that explained the project context and application review process in lay terms was developed. It also highlighted the provision of a £40 "thank you" voucher for attendees. The NIHR CYP MedTech team liaised with the GenerationR YPAG Alliance [11] to circulate the flyer.<br><br>If interested, CYP could follow a link provided in the flyer (in text and QR code form) to an 'expression of interest' form, hosted online via GoogleForms. This form collected minimal information - name, age, email address (of a parent/guardian if under 16) and availability across four potential workshop dates.<br><br>CYP were selected based on the date on which most CYP were available, followed by a first-come-first-served basis up to a maximum of 21 CYP. A waiting list was created for additional CYP.<br><br>Successful CYP were asked to complete an online consent or consent/assent form (for under 16s), then sent the zoom link to join the workshop.<br><br>In planning the workshop, the project team split the attendees into three groups by age, then assigned 2-3 applications to each group, ensuring each group had a good mix of applications (i.e. different topics, different technology types) and aligning applications to appropriate age groups if necessary (i.e. an innovation aimed at secondary school-aged CYP assigned to that age group). The facilitators then made an easy-read summary of each application in a simple form that highlighted the problem, intended audience, technology proposal, etc.<br><br>Each delegate was then sent an PDF by email to help them prepare for the workshop. This included the easy-read summaries of the applications they would be reviewing in their breakout |

| | |
|---|---|
| | rooms, a glossary of useful terms that they might not be familiar with (i.e. co-design) and an overview of the questions we'd be asking them about each application. It was highlighted that this pre-reading was optional and confidential, and that attendees were welcome to ask any questions they had ahead of the workshop.<br><br>The workshop content ran as follows:<br><br>Arrivals: Welcome, overview of the session and introductions to the project team.<br>Scene-setting: A short, visual presentation of how research is usually funded, and how what we are doing today (involving CYP in reviewing applications) is different *and important*.<br>Move to breakout rooms<br>Ice-breaker introductions<br>Review first application; facilitator gives a verbal overview and checks understanding/answers initial questions; Key review questions are copied into the chat and CYP are given 2 mins to consider them; Initial thoughts are shared verbally or via chat; simple illustrated personas are introduced and CYP are asked for feedback based on the persona's needs/preferences; CYP suggest any questions or advice they would give the applicants; all attendees asked to give a simple yes/no or thumbs up/down to answer "Should we fund this project?"<br>Short break<br>Review second and third applications as above<br>Return to the main Zoom room<br>Each facilitator gives a 1 min summary of their group work.<br>GW explains next steps & thanks everyone for their time.<br><br>After the workshop, participants were sent a £40 voucher to thank them for their time. Depending on their preferences, they were also sent a summary of the workshop, a certificate of participation, and/or a letter of recommendation (i.e. to support college applications). |
| 3: Results<br><br>Outcomes—Report the results of PPI in the study, including both positive and negative outcomes | 19 young people participated in the workshop; aged between 12 and 22 years old (average age of 17). 13 young people were female, 5 were male and 1 identified as non-binary.<br><br>CYP were able to engage with the review process including the use of personas to consider the needs and preferences of families from different backgrounds and in different situations.<br><br>Feedback from the CYP covered a range of factors, including the potential impact of the proposed innovations, and highlighting issues that would prevent CYP from engaging with them (i.e. the name of one innovation was very off-putting for some). |

| | |
|---|---|
| | Interestingly, the applications that CYP ranked highest were also the highest-scoring projects from healthcare professional reviewers. |
| 4: Discussion<br><br>Outcomes—Comment on the extent to which PPI influenced the study overall. Describe positive and negative effects | It could be argued that the impact on the outcomes of the review process was minimal, since the CYP's preferences aligned with those of the healthcare professionals. However, the view of the project team is that the input of the CYP added rigour and depth to the review process, particularly as the CYP provided new insights and useful feedback for project teams who weren't successful in this application.<br><br>Moreover, had the opinions of the CYP varied dramatically from the reviews from healthcare professionals, further steps would have been taken prior to choosing the successful applications, for example sharing the anonymised feedback of the CYP with the professional reviewers for further discussion.<br><br>Additionally, the scope of this work was extended through the GenerationR blog post written by one of the young people who attended, available here: https://generationr.org.uk/gosh-ypag-member-zara-attends-national-institute-of-health-and-care-research-nihr-children-and-young-people-cyp-medtech-cooperative-young-person-reviewer-workshop/<br><br>*"It's so important that researchers take young people's thoughts into account, as often we suggest things that adults hadn't even thought of!"* |
| 5: Reflections<br><br>Critical perspective—Comment critically on the study, reflecting on the things that went well and those that did not, so others can learn from this experience | The groups in each breakout room chose to participate in the workshop in different ways. For example, the older groups were more comfortable having their camera switched on and adding their input verbally in a group discussion, whereas some younger participants preferred to take part through the chat function only. This can be challenging to facilitate, but did not prevent groups from being able to share their feedback, and all were able to comment on whether they thought a project should or shouldn't be funded.<br><br>Preparation for this workshop was very time and labour intensive - each funding application needed to be condensed into a one page, easy-read summary suitable for CYP of a range of ages. Whilst each young person was sent 3 of these summaries (matching the projects they would discuss in the workshop), the workshop session itself needed to be planned under the assumption that CYP hadn't had time, or may not remember, the pre-reading, so that all attendees could participate equally.<br><br>We chose to provide enough vouchers to cover the time CYP may have spent on pre-reading prior to the workshop - £40 in total. |

| | |
|---|---|
| | This was included in the workshop advertisement, which may have contributed to the unusually high response rate we received. This led to a lot of administration work in responding to each request to take part and managing a waiting list.<br><br>Several CYP who could and couldn't attend the workshop contacted us to ask to be put on a mailing list for future events. We are currently not set up to do this (due to GDPR and NHS-based host institution guidelines), however it may be useful to explore this in the future. |

## Supplementary File 2: GRIPP2 Short form for Case Study 2: Nell and the Neonatal Unit

| Section and topic | Item |
|---|---|
| 1: Aim(s) of the PPI | Better understand the lived experiences of families with children that were looked after on a neonatal unit (NNU) and embed this into the storylines of an<br><br>• Shape the existing content of the App to suit the needs of different neonatal families e.g. usability, aesthetics, accessibility<br>• Explore how the App could support a range of neonatal experiences in the future and discuss what other storylines should include |
| 2: Methods<br><br>Provide a clear description of the methods used for patient and public involvement (PPI) in the study | A recruitment flyer that explained the purpose and aims of the App in lay terms was created by the Nell and the Neonatal Unit team. It also noted that participants would receive a £20 “thank you” voucher for their time. The NIHR CYP MedTech and Nell and the Neonatal Unit teams circulated the flyer on social media and through their internal networks. The flyer highlighted that the workshop was intended for those with lived experience of the neonatal unit.<br><br>If interested, potential participants could follow a link or use the URL on the flyer to complete an ‘expression of interest’ form, hosted online via GoogleForms. The form collected basic information that would enable the team to contact participants about inviting them to the workshop.<br><br>Participants were selected based on whether they had experience of a neonatal unit and successful candidates were asked to complete an online consent form for themselves and their children. Upon completion, participants were sent a Microsoft Teams meeting link and some resources / guidance before joining the workshop. Prior to the workshop, the parents were asked to download the beta version of the App and spend some time using it with their children.<br><br>The workshop content ran as follows:<br><br>Arrivals: Welcome, introductions to the team and participants.<br>Scene-setting: A brief visual presentation / run through of the App for anyone that wasn’t able to download the App beforehand.<br>First interactive session: The team hosted a discussion around the “function and usability” of the App, for example, how easy/difficult users found navigating various features of the app, what it looked like, and how suitable the pace and tone was.<br>Second interactive session: Next, there was a more in-depth discussion around the “content and engagement” of the App, for example, were the storylines relevant, was it engaging and useful for children, was the information helpful, would they have found this useful in the NNU, what could we add/change, what other storylines should we consider. |

| | |
|---|---|
| | After the workshop, the researchers collated the feedback and passed it on to the App developers so it could be integrated into the App. Each family received a £20 voucher for their time. Additionally, participants were offered a letter of recommendation, and the children were also offered a certificate that acknowledged their participation. |
| 3: Results<br><br>Outcomes—Report the results of PPI in the study, including both positive and negative outcomes | 11 people attended the workshop in total. 6 adults were present (all female, ages unknown but from ranged from approximately 30 years of age to mid 40s) 5 children were present (4 female and 1 male, aged between 5 years and 10 years). Note, not all adults attended the workshop with their children.<br><br>Families provided important feedback:<br>• Function and Usability, including what worked well (i.e. colourful) and what could be improved (i.e. clarifying acronyms/terminology)<br>• Content and engagement, including what worked well (i.e. being useful for both parents and children) and what could be improved (i.e. new story ideas, and additional details to add to make the existing stories more realistic) |
| 4: Discussion<br><br>Outcomes—Comment on the extent to which PPI influenced the study overall. Describe positive and negative effects | Involving parents and their children with lived experience of the neonatal unit significantly influenced the project. Not only did the workshop greatly impact the functionality and content within the App, but it has also encouraged the team to continue to integrate meaningful PPI going forward. The participants at the workshop also highlighted that session itself was really helpful and they enjoyed shaping future resources for families.<br><br>**Some of the changes made so far as a result of the PPI were as follows:**<br>More regional accents included – more voice actors were hired to add variation<br>Added a feature around feeding in collaboration with Medela (company that manufactures breast pumps for NNU)<br><br>**PPI approach and impact shared at conferences:**<br>The team presented at several conferences, including Child Health Technology (CHT2023) and the Royal College of Paediatrics and Child Health (RCPCH) conference in 2023, placing PPI at the centre of the presentations, sharing participant ideas and quotations and detailing planned App refinements arising from this feedback. Together, these engagements highlighted the importance and practical impact of PPI and are encouraging others in the field to adopt similar methods. |

| | |
|---|---|
| | **Direct impact on families at focus group:**<br>The families at the focus group shared that it was great to be part of something that could help others manage the experience better<br>They felt listened to and enjoyed having a space to share their experiences and listen to others that have encountered similar situations<br>The parents also found that using the App, even years after being in the NNU, helped them connect with their children and have conversations they didn't know they needed |
| 5: Reflections<br><br>Critical perspective—Comment critically on the study, reflecting on the things that went well and those that did not, so others can learn from this experience | The workshop was an excellent learning opportunity for the Nell and the Neonatal Unit team and it enabled them to reflect on the process of engaging parents and children in the development of the App.<br><br>One of the strengths of this workshop was that it provided benefits for the participants directly, as well as for those who choose to use the App in the future. The session provided the space for an open discussion where parents and their children could share their thoughts and experiences with people who understood them. It was a positive experience for the families because it allowed them to openly reflect on their past together; it opened up a lot of dialogue with the families and resulted in a meaningful, in-depth discussion about what it's like in a NNU. This led to incredibly helpful conversations about how the App could help people going through these experiences now.<br><br>The participants had lived experience of a NNU and so the App content has been shaped to accurately reflect the experiences of neonatal families. Additionally, the length of stay and the time since participants were discharged from the NNU varied – parents had time to reflect on the experience and see how it shaped their family in the time that followed. As a result, they had a deep understanding of how being in the NNU can impact a family in the longer term<br><br>The recruitment process involved sharing the flyer through social media, the networks of the NIHR CYP MedTech and Nell and the Neonatal Unit teams. There was not a large focus on recruiting those from a wide variety of backgrounds and so the participants were not representative of the neonatal family population. Most participants were females from white / Caucasian backgrounds. Going forward it would be ideal to engage neonatal families from a wider range of socioeconomic backgrounds to ensure the content is relevant to as many people as possible. Currently, the existing storylines and features in the app may not be representative of neonatal families in the UK. As the project progresses and more content is co-developed, the team acknowledges that existing content may need adjusting. For example, language barriers, religious/cultural differences, being younger/older parents, financial situations, non-nuclear family dynamics all may have a previously underestimated impact on someone's experience of a neonatal unit. |

| | |
|---|---|
| | The idea of digital exclusion was briefly covered during the workshop but it would be better in the future to have more emphasis on this vital aspect of digital health. |

## Supplementary File 3: GRIPP2 Short form for Case Study 3: Elixir

| Section and topic | Item |
|---|---|
| 1: Aim(s) of the PPI | To engage with parents and consider their views on the 'Elixir' platform in order to:<br>• De-risk further technology innovation.<br>• Ensure the platform in development fulfils the needs and requirements of parents that will use the technology.<br>• Inform on the design and content of the platform. |
| 2: Methods<br><br>Provide a clear description of the methods used for patient and public involvement (PPI) in the study | The NIHR CYP MedTech team circulated a recruitment flyer via the TITCH network and on social media, that explained the context and aims of the workshop.<br><br>The researchers convened two phases of focus groups with parents which both took place online via Microsoft Teams.<br><br>At the first phase of focus groups the use of the platform and the use of AI to triage and assist with diagnosis in a number of use cases (including in A&E) was discussed.<br><br>At the second phase of focus groups parents were shown a walk through of how the platform would work, and were consulted on topics centred around initial impressions/perceived value, anticipated use and expectations, and concerns and enhancements.<br><br>A third phase of focus groups will involve Healthcare Professionals with experience of working in the emergency department.<br><br>All participants received a £20 gift voucher as a thank you for their time. |
| 3: Results<br><br>Outcomes—Report the results of PPI in the study, including both positive and negative outcomes | The purpose of the PPI sessions was to consult with parents on whether they would use the Elixir platform in a number of health related scenarios, and to gather feedback on how the platform could be altered to improve the user experience/make it more likely that the parents would be comfortable using the platform.<br><br>Using resources pre prepared by the research team and Elixir AI Ltd., participants were given an introduction to the platform and were asked a series of questions.<br><br>In phase 1, topics centred around their thoughts/feelings around sharing potentially sensitive information and images, security of sending/receiving this information, and how to enhance the 'touchpoints' with the service. It was generally accepted that AI could be useful in this context, but work would need to be put in to ensure the 'human touch' is not lost. |

| | |
|---|---|
| | In phase 2, parents were shown a walk-through of how the platform would work, and were consulted on topics centred around initial impressions and perceived value, anticipated use and expectations, and concerns and enhancements. Feedback on ease of use was positive, but concerns were raised on ensuring this did not replace the involvement of healthcare professionals, as well as building trust in the system.<br><br>Participants also repeatedly raised concerns about how other groups, such as an older generation and those who come from difficult socioeconomic backgrounds, may feel when being confronted with this technology, and therefore agreed that this should not be used to replace traditional avenues, and should be optional. |
| 4: Discussion<br><br>Outcomes—Comment on the extent to which PPI influenced the study overall. Describe positive and negative effects | PPI work in this study has successfully influenced the development of the Elixir platform to ensure the needs and requirements of parents has been considered at all levels. Positive feedback received from parents has also been successful in de-risking further innovation.<br><br>Following on from this study, Elixir AI were successful in receiving funding to conduct a multi-centre project to detect biliary atresia, a condition consulted upon in this project. |
| 5: Reflections<br><br>Critical perspective—Comment critically on the study, reflecting on the things that went well and those that did not, so others can learn from this experience | The Elixir platform had already been developed up to a certain point without the input of families. The dashboard could be completely different if work had been conducted earlier, however, it is difficult to deviate too much from a platform as it is and instead only smaller alterations are likely rather than wide scale changes. This however is unlikely to be an issue as the platform was widely very well received by parents.<br><br>As the population was quite broad (anyone who is a parent), it was difficult to recruit for this study as it wasn't specific to anyone. Most recruitment was done using posters, social media and CYP MedTech dissemination networks (e.g. TITCH and newsletters). Therefore the population in attendance were more likely to react positively to the platform as they are already engaged in the healthcare/research environment. In the future it would be beneficial to attempt to include harder to reach populations.<br><br>The age of the participants and their children varied i.e. some parents had newborn babies while others had children in their 20s who had left home. This was a strength of the study as different age groups have different concerns about AI technology and different attitudes to the health services given their differing lived experiences. |
